# 

**Published:** 1920-03-27

**Authors:** 


### Forthcoming Events.

QUEEN CHARLOTTE'S LYING-IN HOSPITAL, N.W.—Annual Meeting of Governors and Subscribers at the Hospital, Tuesday, 30th inst., at 5 P.M.

HEALTH week, which was instituted in 1912, will be held from May 2 to 8, and the central organisation is in the hands of a general committee at the Royal Sanitary Institute, 90 Buckingham Palace Road, S.W.

KING EDWARD'S HOSPITAL FUND FOR LONDON.—His Royal Highness the Prince of Wales has appointed the following persons to be a committee to exercise the powers of President of King Edward's Hospital Fund for London during his absence from the United Kingdom: The Earl of Donoughmore, K.P., the Viscount Finlay, G.C.M.G., and the Governor of the Bank of England.

### The College of Nursing
### (Limited by Guarantee).

### EDINBURGH CENTRE.

ON Tuesday, March 16, Colonel H. Wade, C.M.G., D.S.O., R.A.M.C., gave, by special request, a lecture, entitled "Egypt," to the members of the Edinburgh Centre of the College of Nursing.

The lecture, illustrated by lantern views, proved a great attraction; the seating accommodation of the College room at 8 Drumsheugh Gardens was taxed to its utmost.

The lecturer gave vivid and graphic descriptions of the country, novel modes of travel, and difficulties of transport, especially in the desert regions. His accounts of medical work and hospital administration under war conditions in the country of which he spoke made his hearers realise something of the privations endured and the difficulties met and overcome by doctors, nurses, and men alike at that time.

### LIVERPOOL CENTRE.

AT the meeting on March 17 an interesting lecture was given by Miss Mary H. F. Ivens, M.B., M.S., on "What We Have Learned in the War." The business of the meeting included a report that the American Tea had been a great success. From the proceeds a cheque for £50 has been sent to the College for the Chair of Nursing, and a cheque for 10 per cent. of the surplus for the Endowment Fund of the College. The surplus is to be spent on comforts for the Club Room.

The next lecture will be on APRIL 14, at 7 P.M., in the Lecture Theatre of the Royal Infirmary, on "Orthopædics," by C. B. Alexander, Esq., M.R.C.S., L.R.C.P. Members free; non-members, 6d. per lecture.

### Matrons' and Sisters' Appointments.

HARBOROUGH ROAD HOSPITAL FOR INFECTIOUS DISEASES, NORTHAMPTON.—Miss Ellen Howard has been appointed sister. She was trained at the St. Helens General Hospital, where she was later staff nurse, and held a similar post at the Highfield Military Hospital, Liverpool.

THE OCKENDEN CONVALESCENT HOME, TORQUAY.—Miss Emily Glover has been appointed lady superintendent. She was trained at the Royal Devon and Exeter Hospital, where she has successively held the posts of operating theatre sister and night superintendent, has from time to time undertaken assistant matron's duties, and at present is sister of a male surgical ward.

TORBAY HOSPITAL PROVIDENT DISPENSARY, TORQUAY.—Miss E. M. Adams has been appointed night sister. She was trained at the Birmingham General Hospital and the City Fever Hospital, Birmingham. She has been ward sister at the Kent and Canterbury General Hospital; Stamford General Hospital; Lodge Moor Infectious Diseases Hospital, Sheffield; the London Fever Hospital; Belvidere Hospital, Glasgow; Shoreditch Infirmary; and night sister at the West Bromwich and District Hospital. Miss Adams has also held a post at the Third Northern General Hospital, Sheffield, as a member of the Territorial Force Nursing Service. Miss Adams holds the certificate of the Central Midwives' Board.

ROYAL INFIRMARY, GLOUCESTER.—Miss Edsall Burch has been appointed sister. She was trained at the Royal Cornwall Infirmary, Truro, where she was later sister, and she has also been sister at the Royal Naval Hospital, Plymouth.

THE GENERAL INFIRMARY AT LEEDS.—Miss Gertrude Bulman has been appointed assistant matron. She was trained at the above-named institution, has since been night superintendent and assistant matron at the Royal Infirmary, Derby, sister at the 2nd Northern General Hospital, Leeds, and has been on foreign service from May 1915 to March 1919.

RATES OF SUBSCRIPTION

Twelve months, 10/10; Six months, 5/5; Three months, 2/9, post free to any address in the United Kingdom. Abroad, Twelve months, 13/-; Six months 6/6; Three months, 3/3, post free. **THE HOSPITAL "Index" to Volume LXVI., April to September 1919, is now ready, price 6½d. per copy, post free.** Address The Manager, THE HOSPITAL, "The Hospital" Building, 28/29 Southampton Street, Strand, London, W.C. 2.

